# Defining Continuous Walking Events in Free-Living Environments: Mind the Gap

**DOI:** 10.3390/s22051720

**Published:** 2022-02-22

**Authors:** Abolanle R. Gbadamosi, Benjamin N. Griffiths, Alexandra M. Clarke-Cornwell, Malcolm H. Granat

**Affiliations:** School of Health and Society, The University of Salford, Salford M6 6PU, UK; a.r.gbadamosi@edu.salford.ac.uk (A.R.G.); drjamin1990@gmail.com (B.N.G.); a.m.clarke-cornwell@salford.ac.uk (A.M.C.-C.)

**Keywords:** accelerometers, interruptions in walking, MVPA

## Abstract

In free-living environments, continuous walking can be challenging to achieve without encountering interruptions, making it difficult to define a continuous walking event. While limited research has been conducted to define a continuous walking event that accounts for interruptions, no method has considered the intensity change caused by these interruptions, which is crucial for achieving the associated health outcomes. A sample of 24 staff members at the University of Salford were recruited. The participants wore an accelerometer-based device (activPAL™) for seven days continuously and completed an activity diary, to explore a novel methodological approach of combining short interruptions of time between walking events based on an average walking cadence. The definition of moderate-to-vigorous physical activity (MVPA) used was a minimum walking cadence of either 76, 100, or 109 steps/min. The average daily time spent in MVPA increased from 75.2 ± 32.6 min to 86.5 ± 37.4 min using the 76 steps/min, 48.3 ± 29.5 min to 53.0 ± 33.3 min using the 100 steps/min threshold, and 31.4 ± 20.5 min to 33.9 ± 22.6 min using the 109 steps/min threshold; the difference before grouping and after grouping was statistically significant (*p* < 0.001). This novel method will enable future analyses of the associations between continuous walking and health-related outcomes.

## 1. Introduction

Sedentary behaviour and physical activity are often reported as total daily time spent at a specific intensity, typically minutes or hours per day. While the volume of these behaviours is important for health [1,2], some recent evidence also suggests that the way these variables are accumulated, such as the length, intensity, and duration of bouts, may be of public health importance [3,4,5]. For example, the 2011 UK physical activity guidelines suggest that adults should participate in at least 150 min of MVPA per week, in continuous bouts of 10 min: these guidelines highlight the volume, intensity, and length of bout required to achieve optimum health benefits [6]. The 2011 guidelines were updated in 2019 to include every bout of walking, irrespective of the length of the bout [7].

Continuous walking can be interrupted by a pedestrian stopping for moving traffic, waiting for the pedestrian lights, or simply stopping to catch one’s breath [8,9]. Monitoring continuous walking is important for understanding its impact on health outcomes [10,11]. Currently, there is no standardised definition for a continuous walking event [9]; however, some activity monitoring devices (the ActiGraph) may define continuous walking as a single episode of stepping with the assumption that walking was continuous in that minute and not taking into account any interruptions of sitting or standing [12]. Daily walking can be achieved within a range of intensities that are important to consider because of the associated health benefits. MVPA of 10 min or more has been found to reduce the risk factors for cardiometabolic disease and metabolic syndrome [13]. There is also evidence that bouts of MVPA embedded within bouts of light-intensity physical activity (LIPA) can have similar health benefits [14,15]. Robson and Janssen (2015) suggested that combining short bouts of MVPA, fewer than 10 min in duration, within bouts of LIPA is as beneficial as a continuous bout of MVPA. However, it is not clear from these studies how to robustly combine the different activity intensities. Potentially, incorporating interruptions and varying intensities within the definition of a continuous walking event may be beneficial, while providing similar health benefits.

Alternative definitions of a continuous walking event have already been proposed to account for interruptions in stepping. These studies have focused on defining the minimum interruption duration (typically 6 to 50 s), irrespective of walking bout length or walking intensity [8,15,16]. These interruptions can include quiet standing but not sedentary interruptions, such as sitting or lying down. However, these definitions overlook the intensity of the activity, which is important for the associated health benefits. Cadence has been suggested as a practical way of estimating walking intensities [17,18] and is simple to measure using common activity monitoring devices. Using cadence as a proxy for physical activity intensity, along with the duration of the combined interruption and stepping, could provide a more robust definition of a continuous walking event.

In this study, we explored a new method for defining a walking event based on the intensity of the walking and the duration of the interruption. MVPA has been commonly defined, in healthy adults, as a walking cadence of 100 steps/min [17,18,19,20]; however, other studies have defined MVPA as a cadence of 109 steps/min derived from a laboratory-based study by Tudor-Locke et al. [9,12,21]. In addition, the average cadence of 76 steps/min was reported by Dall and colleagues in a healthy population in a free-living setting [12]; also, the activPAL™ software provides an indirect estimate of METs based on steps using an inbuilt cadence linear regression equation [22] that works out that three METs (moderate intensity) would be approximately 74 steps/min. Therefore, the 76 steps/min threshold was considered a cadence threshold for MVPA and a reference point to investigate the effect of combining walking events compared to other established thresholds. Therefore, these three cadence thresholds were used to combine walking bouts and redefine continuous walking. We then investigated how this approach affected time spent in MVPA and compliance to the 2011 and 2019 UK physical activity guidelines.

## 2. Methods

### 2.1. Participants

Participants were a convenience sample of staff from the School of Health and Society, University of Salford. Participants were aged 18 years and above, healthy, and with no mobility problems. Twenty-seven participants completed the study; however, three participants were excluded because they did not have complete sleep and waking hours data. All participants gave informed consent, and the study was approved by the University of Salford’s School of Health and Society Research Ethics Panel (HST1617-202).

### 2.2. Data Collection

**Figure 1:**
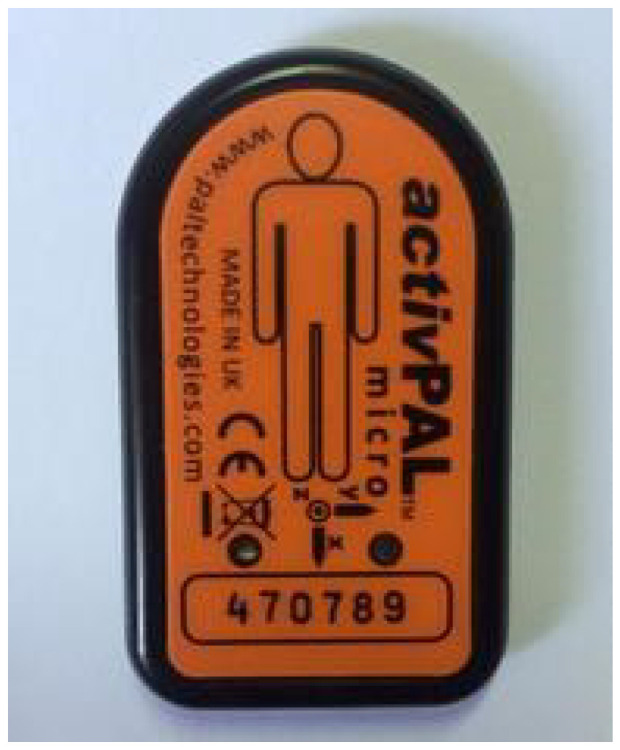
The activPAL™ micro accelerometer, with the figure on the front indicating the direction of attachment.

Free-living activities were assessed using the activPAL™ activity monitor (Figure 1) [23]. The activPAL™ is a thigh-worn activity monitor and its attachment to the thigh derives postural classifications based on the inclination along the three orthogonal planes using proprietary algorithms [24]. It provides a valid measure of time spent sitting/lying, standing, and stepping, on a second-by-second basis in a range of populations; children [25], healthy young adults [26,27,28,29], and older adults [30]. This device was used in this study because it provides a robust measure of step count: the second-by-second downloadable events file produced from the proprietary algorithm software classifies all steps that are taken and the duration in which the steps are taken; therefore, the cadence can be calculated [26,31].

The monitor was attached to the front of the thigh mid-way down the limb using a water-resistant dressing, Tegaderm, and was worn continuously for seven days, except when bathing or swimming. On the return of the monitors, the data were downloaded using activPAL™ proprietary algorithms. The output from the device was a time-stamped classification of events as either sitting/lying, standing, or stepping [24]. These event files were downloaded and visually examined to ensure complete data (sleep and waking hours) for each participant. Incomplete days (fewer than 24 h a day) were manually removed at the start and end of the recording period.

### 2.3. Data Processing

**Figure 2:**
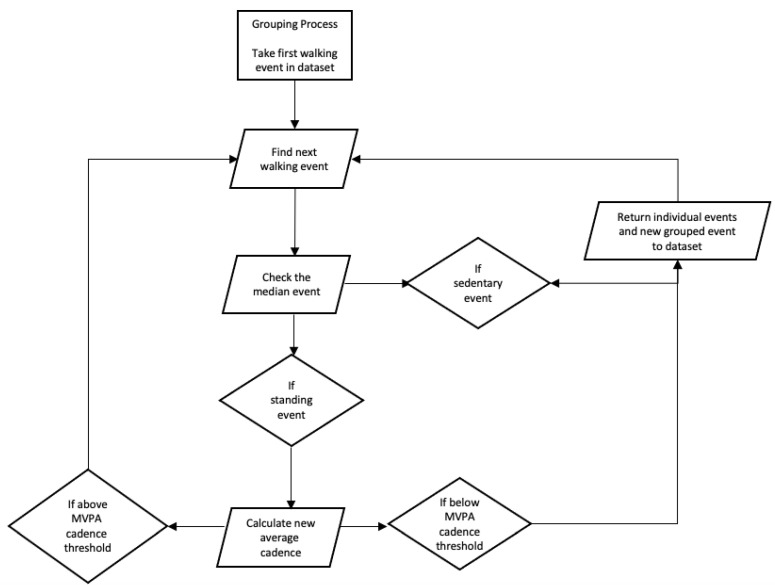
Flow diagram of the data processing method.

The data were processed using MATLAB^®^ (MathWorks Inc., Natick, MA, USA). First, walking events were created by combining consecutive stepping events, and from these, start time, duration, number of steps, and cadence were calculated (Figure 2). This process was performed three times using each of the MVPA cadence thresholds of 76, 100, and 109 steps/min as the defined cadence threshold for grouping, creating three separate datasets.

To redefine a continuous walking event, two consecutive walking events interspersed with a standing event were combined into one new event. Using the total duration and step count for this new event, average cadence was calculated and compared to the defined cadence threshold. If the new event’s average cadence was higher than the defined cadence threshold, the processing would continue and add the next interruption and walking event to the new event. However, if the new walking event’s average cadence fell below the defined cadence threshold, or if the processing encountered a sitting or lying event, the processing would end and the walking event up to this point would be added to the dataset. This process was repeated until all the walking events within the dataset had been analysed.

### 2.4. Data Analysis

The average daily walking duration for the ungrouped (original walking events without interruptions) and three grouped walking event datasets (76, 100, and 109 steps/min) were compared using a repeated measure one-way ANOVA. Means, standard deviations, and percentage increases are presented along with significance level, with significance taken as *p* < 0.05. An analysis of MVPA was conducted for each of the three MVPA cadence thresholds: the average daily time spent in MVPA was calculated using the three cadence thresholds before and after grouping. The defined cadence threshold used for the grouping was also the threshold used to define MVPA. These data were analysed using a repeated measures two-way ANOVA to understand the effects of grouping and MVPA threshold on time spent in MVPA. The composition of the walking events within each of the datasets was analysed by data categorising the walking events into different lengths of walking events based on their total duration. Furthermore, the grouped walking events’ composition was analysed by categorising the original events that made up the grouped events into standing, walking events shorter than the total minimum value of the event bin, and walking events within the same range duration as the event bin. This showed the composition of the new grouped event in terms of standing time, short walking time, and longer walking time.

## 3. Results

### 3.1. Descriptive Analyses

**Figure 3:**
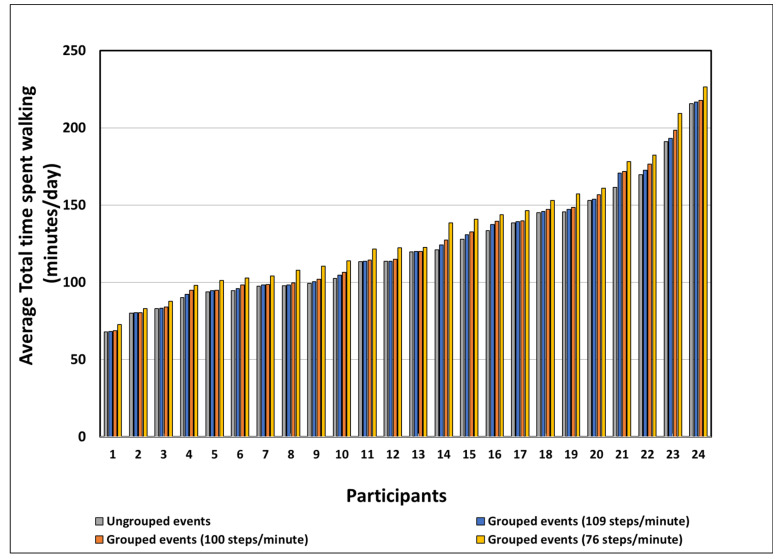
The average total duration of walking per day between ungrouped walking events and grouped walking events at each defined cadence threshold (participants are presented in increasing order according to ungrouped events).

The average time spent walking before grouping (123.1 ± 35.8 min) was less than the average time spent walking after grouping at all defined cadence thresholds. The average time walking using the 76 steps/min (132.7 ± 38.3 min) was greater than the average time spent walking using the 100 steps/min threshold (126.3 ± 37.2 min) and the 109 steps/min threshold (124.7 ± 36.6 min) (Figure 3). Figure 3 presents the average time spent walking for all participants in increasing order according to the ungrouped events. After grouping, the average time spent walking increased by 8% for the 76 steps/min threshold, 3% for the 100 steps/min threshold, and 1% for the 109 steps/min threshold. All the changes were significant (F (1.5, 36.1) = 123.6, *p* < 0.05), and the post hoc pairwise comparisons using the Tukey test showed significant differences between all the datasets (*p* < 0.05).

### 3.2. Time in MVPA

**Figure 4:**
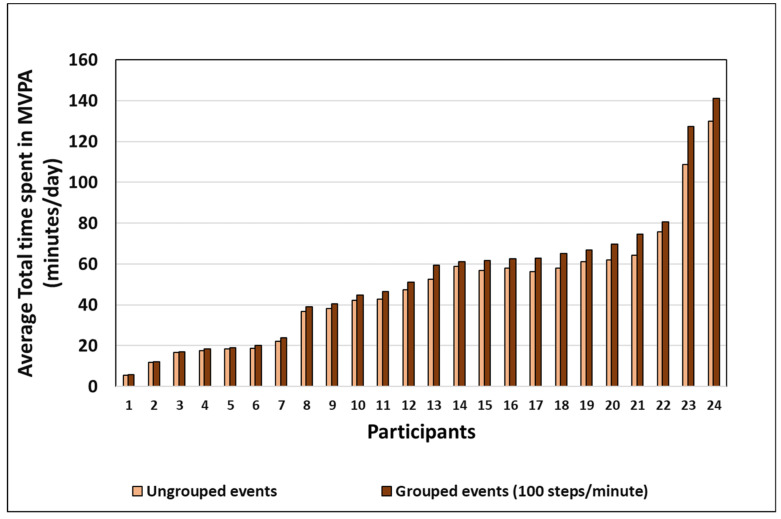
Total time in MVPA walking at 100 steps/min per day for all participants.

Figure 4 shows the average daily time spent walking at MVPA for the ungrouped data and the 100 steps/min defined cadence threshold data, the most common MVPA threshold. Grouping increased the total time spent in MVPA to between 2% and 17% (0.3 min to 11.2 min) depending on the MVPA time accumulated by the participant. The average daily time spent in MVPA increased from 75.2 ± 32.6 min to 86.5 ± 37.4 min using the 76 steps/min, 48.3 ± 29.5 min to 53.0 ± 33.3 min using the 100 steps/min threshold, and 31.4 ± 20.5 min to 33.9 ± 22.6 min using the 109 steps/min threshold. The results of the two-way repeated-measures ANOVA revealed that there was a significant main effect of the threshold on time spent in MVPA at 76 steps/min (F (1.4, 31.1) = 175.8, *p* < 0.05, ηp^2^ = 0.884). Similarly, there was a significant main effect of the grouping process on time spent in MVPA at 100 steps/min (F (1, 23) = 61.2, *p* < 0.05, ηp^2^ = 0.727) and a significant interaction between the defined cadence threshold and the grouping process (F (1.6, 36.8) = 83.4, *p* < 0.05, ηp^2^ = 0.784), such that the increase in time spent in MVPA through grouping was related to the choice of MVPA threshold. Again, post hoc pairwise comparison using the Tukey test showed significant differences between all the datasets (*p* < 0.05).

### 3.3. Lengths of Walking Events

**Figure 5:**
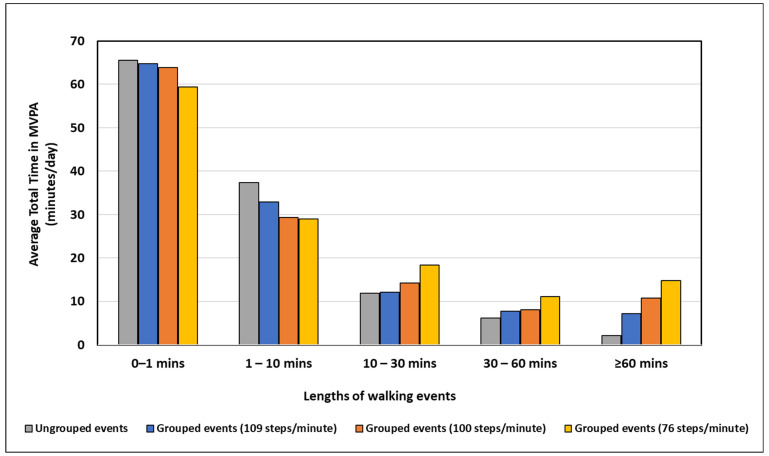
Walking events lengths of ungrouped and grouped events in terms of duration per day.

Figure 5 shows the distribution of the walking event lengths at the different defined cadence thresholds. Grouping resulted in a redistribution of short-duration walking events to longer-duration walking events, with this being more pronounced in the shortest and longest walking event lengths. For walking events that lasted <1 min, there was an average duration of 65.5 min across all participants per day in the ungrouped data, compared to 63.9 min for the 100 steps/min defined cadence threshold, showing that these events were being incorporated in longer events as part of the grouping process. For walking events >60 min, there was an average duration of 2.2 min across all participants per day in the ungrouped data and 10.8 min for the 100 steps/min defined cadence threshold, showing that the grouping process increased the amount of long continuous walking. This shift from short-duration walking events to longer-duration walking events was also prominent in the 76 steps/min (59.4 min for <1 min and 14.8 min for >60 min) and 109 steps/min defined cadence threshold (64.8 min for <1 min and 7.2 min for ≥60 min).

### 3.4. Composition of the Events Included in the Grouping Analysis

**Figure 6:**
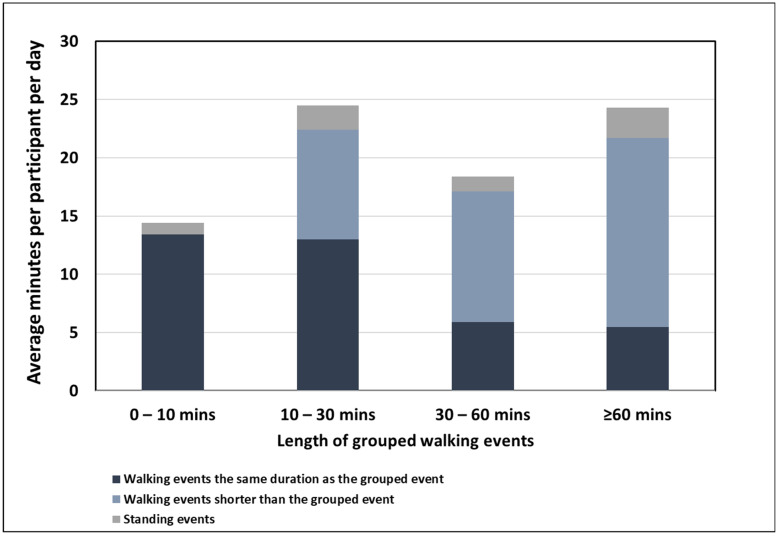
Composition of grouped events where each bar shows the composition of walking events within each event length category.

Figure 6 shows the composition of the grouped walking events using the 100 steps/min defined cadence threshold for four different lengths of walking events. The shorter walking duration of <10 min was exclusively made up of walking events equal to this duration and short standing events; no events shorter than this duration were included as this was the smallest possible duration range. As the duration of walking event length increased, the distribution of walking events that make up the event length shifted predominantly from events of the same duration to events shorter than the duration. This shows that the longer walking duration is predominantly made up of shorter events and highlights the influence of the grouping process on these bout lengths that would be considered continuous walking (≥10 min).

At the typical MVPA cadence threshold of 100 steps/min, the standing events that were included in the grouping process ranged from 0.9 to 2.5 min in length, whereas at 76 steps/min, the standing events ranged from 1.9 to 5.1 min long, and at 109 steps/min, the standing events ranged from 0.5 to 1.8 min.

### 3.5. Compliance with Physical Activity Guidelines

Comparing the participants’ activity levels against the 2011 physical activity guidelines where at least 30 min of MVPA per day and a minimum bout length of 10 min or more is stipulated (MVPA defined as ≥100 steps/min), only seven participants were compliant with the guidelines before grouping; however, after grouping, seven additional participants met the guidelines. With the 2019 guidelines (at least 30 min of MVPA per day on five days a week, with no restriction of bout lengths), there were 17 participants before grouping, with an additional three meeting the guidelines after grouping.

## 4. Discussion

This is the first study to use an intensity-based approach to define continuous walking events. Combining walking events with interruptions and assessing the intensity of the new walking bout using an MVPA defined cadence threshold: this method does not make any prior or arbitrary decision on the length of the walking bout or the interruption. The intensity-based approach can be used as a robust definition of continuous walking that may reflect health benefits associated with this physical activity.

The grouping method showed a significant increase in average daily walking duration for all participants for both total accrued walking and walking at MVPA intensity. This increase was significant across all three defined cadence thresholds, demonstrating the benefit of using this method for assessing time spent in MVPA using a range of established thresholds. Furthermore, it highlights the impact that short interruptions are having on the assessment of time spent in MVPA, which is likely impacting the assessment of health outcomes and physical activity prescription.

The overall finding of this study is similar to Barry et al. [8], which showed an increase in the total volume of activity accumulated by participants when short interruptions were considered. However, the authors proposed a maximum length of interruption and only reclassified standing events as walking events if they fell below this time threshold. The method could incorporate interruptions equal in length to the corresponding walking events and does not consider how this might impact the change in intensity of the total walking event. Using cadence as a proxy for intensity when combining walking events with interruptions reflects the physiological processes associated with continuous physical activity [16] and is more likely to be associated with the health benefits of this activity.

The grouping process changed the composition of the events within each dataset, with the largest percentage change being seen in events longer than 60 min in duration. Continuous walking longer than 60 min increased by 200% for the 100 steps/min, and 500% at the 76 steps/min threshold. However, these changes can also be seen in events lasting longer than 10 min in duration: these longer walking events were predominantly composed of a combination of short and medium-length walking events, with a small proportion of this time coming from the inclusion of standing events. This demonstrates that in free-living environments, there are few opportunities for walking continuously for long periods without interruption and highlights the need for a robust definition of continuous walking.

Before grouping, seven of the 24 participants met the 2011 guidelines, while 17 met the 2019 physical activity guidelines. After grouping, the number of participants meeting the 2011 guidelines increased to 14 (100% increase), while the number of participants meeting the 2019 guidelines increased to 22 (18% increase). We hypothesise that in addition to the evidence regarding the impact of walking bouts less than 10 min being beneficial to health, another reason for the update on the 2011 UK guidelines could be partly due to the difficulty in the definition of continuous MVPA and that continuous walking bouts of 10 min was not practical in free-living settings. For both 2011 and 2019 guidelines, combining walking events can significantly impact levels of compliance and the presented grouping method could enable a reintroduction of a minimum bout length, given it reflected an associated health benefit. The removal of the minimum bout length requirement may give a false representation of those meeting up with guidelines; however, standardised quantification of the dimensions of physical activity (mode, frequency, duration, and intensity) is vital to assessing compliance to physical activity levels.

The grouping process may also be useful for assessing impaired walking populations where it would not be appropriate to use the same MVPA threshold used for a healthy nonimpaired population. For example, it has been shown that people with intermittent claudication walk with a slower cadence than matched healthy controls [32], and perhaps the chosen cadence threshold should be altered to enable this population to engage in continuous walking. The method presented provides a simple way of combining walking events based on the cadence threshold suitable for different populations, and future work should aim to derive these cadence thresholds and establish their impact on health outcomes.

## 5. Conclusions

This study presents a robust and practical methodology for defining continuous walking events to help understand the impact of continuous walking on health outcomes. The technique groups walking events with short interruptions based on their average cadence, comparing this to a defined cadence threshold. This considers the intensity of the walking bout, compared with existing methods that set a specific maximum interruption threshold based on time. After applying the grouping to a dataset of healthy participants, the average time spent walking and walking at MVPA significantly increased. This technique can be easily adapted to suit other populations with walking impairment and may enable this group to engage in continuous walking and meet physical activity guidelines.

## Data Availability

The data presented in this study are available on request from the corresponding author. The data are not publicly available due to privacy.
